# Ethnic Differences in Abstinence Self-Efficacy among Recovering Individuals

**DOI:** 10.17352/2455-3484.000015

**Published:** 2016-12-02

**Authors:** Elbia Navarro, Josefina Alvarez, Richard Contreras, Leonard A Jason

**Affiliations:** 1 DePaul University, USA; 2 Adler University, USA; 3 Healthcare Alternative Systems, USA

**Keywords:** Abstinence self-efficacy, Addiction, Recovery, Ethnicity

## Abstract

The purpose of the current study was to explore ethnic differences in drug abstinence self-efficacy among recovering individuals. Levels of abstinence self-efficacy among African Americans and European Americans increased, decreased, and then increased again over the year. Drug abstinence self-efficacy remained stable over time among Latinos in this study. It is possible that, although they have reported positive experiences in Oxford House, Latinos may not receive the same benefits other groups gain from participation in Oxford House. Another possible explanation for the lack of change in abstinence selfefficacy among Latinos in Oxford House may be that factors outside the house and support networks may lower expectations for recovery. These factors may include inability to find work, experiences of discrimination, or lack of access to care. Future research needs to explore the social networks of Latinos in Oxford House as well as the experiences of this ethnic group in this program and in the community.

## Introduction

### Ethnic Differences in Abstinence Self-Efficacy among Recovering Individuals

According to the 2010 Census, Latinos comprise 16.3% of the total U.S. population. This group continues to grow and by the year 2050, Latinos are expected make up 25% of the total U.S. population [1]. National surveys indicate that this group engages in substance use behavior at similar rates to the national average [2], but have higher rates of substance-related problems compared to other groups [3]. Current research indicates that Latinos underutilize substance abuse treatment [4–6], but the reasons for this groups’ treatment underutilization are not well-understood.

Socio-demographic characteristics are associated with substance abuse treatment utilization among Latinos. In particular, individuals who are younger, have lower incomes and who have no health insurance are least likely to use mental health and substance use services [7]. English speaking and U.S. born Latinos are more likely to utilize substance abuse treatment compared to their immigrant and Spanish-speaking counterparts [8,9].

In regards to treatment outcomes, there are mixed findings. Some research indicates that Latinos are more likely to drop out of treatment and to report that their needs were not met than other ethnic groups [10]. Other research indicates that European Americans compared to Latinos were more likely to remain abstinent after completing substance abuse treatment [11,12]. Other studies have shown similar outcomes for Latinos and other ethnic groups [13–16]. Finally, research supports different treatment outcomes among Latino subgroups and suggests that a number of individual characteristics may predict treatment outcomes among Latinos [17]. There is a need to continue to explore factors related to utilization and outcomes for Latinos in need of substance abuse treatment [17,18].

Exploring abstinence self-efficacy may help elucidate ethnic disparities reported in the substance abuse literature. Abstinence self-efficacy is defined as one’s belief that one will not engage in substance use when confronted with situations that can trigger relapse [19–21]. Studies have shown that abstinence self-efficacy plays a vital role in the maintenance of abstinence from drugs and alcohol in several research studies. For example, Walton and colleagues [22] found that abstinence self-efficacy predicted alcohol relapse. In another study, individuals with substance use disorders who received treatment and had higher levels of confidence in abstaining from drugs and alcohol were more likely to remain abstinent for a year [19]. Other research has similarly shown that abstinence self-efficacy predicts lower rates of alcohol and cocaine use as well as the stability of long-term recovery [21,23].

Based on the review of the literature, there is only one study that has extensively looked at self-efficacy among Latinos in treatment for substance abuse. In this study, among a sample of Latinos in methadone maintenance, those with lower levels of self-efficacy had higher levels of poly-substance use at the 12-month follow-up [24]. Other studies have shown that among individuals with substance abuse and psychiatric disorders, African Americans demonstrated higher levels of abstinence self-efficacy compared to European Americans and Latinos [22,21].

Oxford House is a program that operates community based self-run recovery homes. Oxford House residents are required to pay their equal share of the rent, contribute to the maintenance of the house and abstain from substance use [25]. The effectiveness of Oxford Houses among individuals in recovery has been shown in several studies [26–28]. Additionally, individuals who lived in Oxford Houses for longer than 6 months were more likely to remain abstinent and to report higher levels of abstinence self-efficacy [27]. Finally, Oxford House residents are more likely to be confident that they will remain abstinent from substance abuse compared to individuals who participated only in Alcoholics Anonymous groups [29].

Although research has shown that Oxford House can be effective for individuals in recovery, Latinos are not well-represented in Oxford Houses [27]. In spite of the underrepresentation of Latinos in Oxford Houses, Latinos who have resided in Oxford Houses have mentioned that these communal settings have given them a positive and supportive recovery environment [30]. The current study sought to explore ethnic differences in abstinence self-efficacy among Latinos, African Americans and European Americans in Oxford House.

## Methods

### Participants and Procedures

This study used a dataset from a larger investigation on Oxford Houses [27]. The majority of participants were recruited from 170 Oxford Houses in the following states: Washington, Oregon, Pennsylvania, New Jersey, North Carolina, Illinois, and Texas. Other participants were recruited at an Oxford House World Convention. Research assistants administered the questionnaires to participants in person after explaining to them the purpose of the study and signing informed consent [27]. Data were collected every 4 months in the one-year longitudinal study.

There were 897 participants in the original study and of these, only 31 participants were of Latino ethnicity. The demographics of Latino Oxford House residents are as follows: 23 Latino participants were males and 7 Latino participants were females. Twenty-three Latino participants were born in the United States, 7 were born outside of the United States, and 1 person did not provide information about their birthplace. Out of 7 Latino participants who were born outside of the U.S., only three provided information about their length of stay in the U.S. One Latino lived here in the United States for 17 years, the other said for 27 years, and the third person said for 32 years. Out of 31 Latino participants, 21 Latinos said they spoke at least some Spanish.

Since the purpose of the study was to explore ethnic differences in self-efficacy among Oxford House residents, 31 Latino participants were matched with 31 European Americans and 31 African Americans based on age, gender, and years of education revealing a total of 93 participants in the current study. The mean age in years of the entire sample was 33.9 (SD=8.8). The mean years of education were 11.5 years (SD=2.0). The mean months of residency in Oxford House was 9.1 (SD=14.97). One-way analyses of variance (ANOVA) revealed that there were no significant ethnic differences in age or level of education for the current sample. In addition, there were no significant ethnic differences in length of residency in Oxford Houses.

### Measures

*The Addiction Severity Index-lite (ASI-lite)* McLellan et al., 1992 is a valid and reliable measure that gathers information on demographics, history of treatment use, lifetime and 30 days substance use, and problem severity in the medical, psychological, family, employment, and legal domain. Problem severity in each of these domains is based on a composite score, which has been shown to be valid and reliable. In the current study, the ASI was used to gather information about the participant’s age, gender, years of education, their length of stay as residents of Oxford House, and their lifetime substance use.

*The TimeLine Follow-back (Form-90),* Miller and Del Boca, 1994, was used to collect data on the number of days participants used substances in the past 90 days. Form-90 has good reliability on measuring the number of days of alcohol use and a moderate reliability on measuring the number of days of drug use. The current study used data on past 90 day illicit drug use of the participant to analyze whether there were any significant ethnic differences in past 90 day drug use at baseline and at follow-up.

*The Drug Abstinence Self-Efficacy Scale (DASE)* is a slightly modified version of the Alcohol Abstinence Self-Efficacy Scale (AASE) consisting of 20 items that asks participants to rate their confidence level of abstaining from alcohol in situations that can trigger relapse to substance use. Words “drink alcohol” were replaced with “use drugs” in order to capture the confidence level of participants in abstaining from situations that involve drug use. Answering questions from the DASE was based on a Likert scale (1=not at all confident, 5= extremely confident). Lower drug abstinence self-efficacy scores indicated low self-efficacy in abstaining from drug use. High drug abstinence self-efficacy scores indicated high self-efficacy in abstaining from drugs. The current sample’s Cronbach’s alpha for the DASE was .99.

## Results

### Lifetime and 90 day substance use

Table 1 shows the means for lifetime substance use for each ethnic group. A MANOVA was used to examine whether there were any significant ethnic differences for years in drug use. Significant ethnic differences were found in years of heroin use [F(2,88)= 3.65, *p* <.05], amphetamine use [F (2,88)=4.99, *p* <.05], and hallucinogen use [F (2,88)=5.93, *p* <.05]. Tukey HSD post hoc tests indicated that European Americans had significantly more years of amphetamine use than African Americans (*p* <.05) and more years of hallucinogen use than African Americans (*p* <.05) and Latinos (*p* <.05). Latinos had more years of heroin use than African Americans (*p* <.05).

Table 2 shows the means for past 90 day drug use at baseline and follow-up measurements. An ANOVA was used to examine whether there were any significant ethnic difference in past 90 day drug use. Multivariate tests indicated that there were no significant ethnic differences for past 90 day drug use at baseline and at follow-up measurements.

### Baseline ethnic differences in drug abstinence self efficacy

Table 3 shows the means and standard deviations for drug abstinence self-efficacy for each ethnic group at baseline and at the one-year follow-up. An analysis of covariance (ANCOVA), controlling for length of residency in Oxford House, revealed that there were significant ethnic differences in drug abstinence self-efficacy [F (2, 89) = 3.69, *p* <.05]. Tukey HSD post hoc tests indicated that Latinos had significantly lower drug abstinence self-efficacy than African Americans (p. <.05). There were no significant differences in drug abstinence self-efficacy between Latinos and European Americans.

### Longitudinal changes in abstinence self-efficacy

Hierarchical Linear Modeling HLM; Bryk, Raudenbush, & Congdon, 2004, was used to examine whether the pattern of the DASE scores across the three ethnic groups were different from each other during the one year longitudinal study. This is advantageous, in that all data from participants may be used, even if they have not completed DASE assessments at each time point. Specifically, the linear, quadratic, and cubic trend were entered into the model. Furthermore, dummy coded variables were entered such that Latinos were the reference group and both (1) Caucasians and (2) African-Americans were compared to Latinos and were entered as Level 2 predictors. The following HLM model was estimated:

$$\begin{array}{l} \text{Level}\;1:Y_{\text{ij}}=\beta_{0i}+\beta_{1i}\;{\text{Time}}_{i}+\beta_{2i}\;{{\text{Time}}_{i}}^{2}+\beta_{3i}\;{{\text{Time}}_{i}}^{3}+r_{\text{ij}}\\ \text{Level}\;2:\beta_{0i}=\gamma_{00}+\gamma_{01}\;\text{Caucasian}\;\text{vs.}\;\text{Latino}+\gamma_{02}\;\text{African}-\text{American}\;\text{vs.}\;\text{Latino}+u_{o}\\ \beta_{0i}=\gamma_{00}+\gamma_{01}\;\text{Caucasian}\;\text{vs.}\;\text{Latino}+\gamma_{02}\;\text{African}-\text{American}\;\text{vs.}\;\text{Latino}\\ \beta_{0i}=\gamma_{00}+\gamma_{01}\;\text{Caucasian}\;\text{vs.}\;\text{Latino}+\gamma_{02}\;\text{African}-\text{American}\;\text{vs.}\;\text{Latino}\\ \beta_{0i}=\gamma_{00}+\gamma_{01}\;\text{Caucasian}\;\text{vs.}\;\text{Latino}+\gamma_{02}\;\text{African}-\text{American}\;\text{vs.}\;\text{Latino} \end{array}$$

Latinos DASE scores differed significantly from zero at baseline, γ = 3.72, t(90) = 16.73, p < .001. There was no statistically significant difference between Latinos and Caucasians at baseline, γ = 0.37, t(90) = 1.27, p = .21. However, there was a statistically significant difference between Latinos and African-Americans, such that African-Americans scored higher on the DASE initially than Latinos, γ = .72, t(90) = 2.70, p = .009. Latinos did not experience a statistically significant linear, quadratic, or cubic time trend. In other words, their scores were stable over time. Caucasians differed marginally from Latinos in their linear trend (γ = 1.76, t(276) = 1.92, *p* < .06), such that their scores were increasing over time, but they also experienced significant differences in their quadratic (γ = −2.24, t(276) = −2.52, *p* < .01) and cubic (γ = 0.53, t(276) = 2.64, *p* < .01) trends, such that they experienced no change initially, followed by a decrease and then an increase again. African-Americans differed significantly in their linear trend from Latinos (γ = 3.07, t(276) = 3.15, *p* < .01), but they also differed significantly in their quadratic (γ = 3.52, t(276) = 3.34, *p* <.001) and cubic (γ = 0.81, t(276) = 3.27, *p* < .01) trends. African-Americans experienced an initial increase in DASE scores, followed by a decrease and then an increase again. In addition to the fixed effects described above, we also examined random effects, but only a random intercept was statistically significant, σ^2^ = 0.34, χ^2^ (df =90) = 177.69, *p* < .001. The intra-class correlation coefficient (i.e., ρ, which is calculated by τ / τ + σ^2^), which tells the proportion of variance in the outcome that is due to person characteristics, was .24.

## Discussion

The purpose of the current study was to explore ethnic differences in drug abstinence self-efficacy among recovering individuals. Drug abstinence self-efficacy in Latinos and European American Oxford House residents were not significantly different at baseline. These findings are surprising based on past research studies that indicated higher likelihood of relapse among Latinos compared to European Americans [12,31]. A potential explanation for the lack of significant differences in abstinence self-efficacy may be that the majority of Latino Oxford House residents in the current sample spoke English and were born in the United States. Past studies have shown that compared to Spanish-speaking Latinos, English-speaking Latinos tend to have similar views on social values and future plans to the general U.S. population [32]. Further research may want to explore whether English-speaking Latinos and European Americans share similar views in recovery and whether it is related to abstinence self-efficacy in drug use. Future studies may also want to include a larger more heterogeneous sample that includes English and Spanish speaking Latinos who are both U. S. and foreign born.

The finding that baseline drug abstinence self-efficacy among African Americans was significantly higher than among Latinos is consistent with prior research showing higher levels of abstinence self-efficacy in African Americans than in other ethnic groups [21]. Researchers postulate that accessing to treatment services might have improved their abstinence self-efficacy [21]. In Oxford House, individuals build social support networks which helps individuals increase their self-efficacy in abstaining from drug use [27]. It may be that for African Americans in this study, becoming involved in community resources, such as Oxford House, has helped them feel more confident in abstaining from drug use. Future research exploring access to abstinent support and treatment resources among African Americans and Latinos may shed light on the experiences that may predict the higher levels of abstinent self-efficacy among African American Oxford House residents.

In the current study, levels of abstinence self-efficacy among African Americans and European Americans increased, decreased, and then increased again over the year. According to past research on Oxford Houses, individuals who lived in Oxford House for more than 6 months show increases in abstinence self-efficacy [27]. Oxford House residents encourage each other to work on their recovery, attend 12-step meetings and obtain employment. After staying in Oxford House, individuals build abstinent social support networks during their time living in Oxford House which can influence their self-efficacy [27]. Further research is needed to examine factors that contribute to fluctuations in abstinence self-efficacy while in recovery.

Drug abstinence self-efficacy remained stable over time among Latinos in this study. It is possible that, although they have reported positive experiences in Oxford House, Latinos may not receive the same benefits other groups gain from participation in Oxford House. Another possibility may be that over time cultural and environmental factors might have influenced abstinence self-efficacy among Latinos. According to Alvarez and Ruiz [33], “familismo” includes maintaining close relationships with family members. In one survey, eighty-nine percent of Latinos stated that their families are more important to them than their friends compared to 67% of European Americans and 68% of African Americans [32]. Additionally, Latino substance abusers report that their drug use occurred when their family members were using drugs [34]. Moreover, Latinos were more likely to report using drugs with their family members compared to European Americans and African Americans [34]. Family members who use drugs as part of their daily lives may put Latinos in a position where they will not be receiving support for their recovery. Another possible explanation for the lack of change in abstinence self-efficacy among Latinos is Oxford House may be that factors outside the house and support networks may lower expectations for recovery. These factors may include inability to find work, experiences of discrimination, or lack of access to care. Recent research indicates that perceived barriers to opportunities predict substance use among Latinos [35,36]. Future research needs to explore the social networks of Latinos in Oxford House as well as the experiences of this ethnic group in this program and in the community.

The current study included a sample of 90 participants at baseline and only 63 participants remained at follow-up. In addition, the majority of the Latino participants was English speaking and mostly born in the United States. The results of the current study cannot be generalized to Spanish-speaking Latinos and Latinos who were born outside of the United States. Future research with larger, more diverse samples of Latinos is needed to shed light on the potential predictors of abstinence self-efficacy and treatment outcomes among Latinos in recovery. Further research may want to look at other cultural variables, such as ethnic identity and acculturation, when looking at drug abstinence self-efficacy in Latinos.

In conclusion our study found that drug abstinence self-efficacy in Latinos and European American Oxford House residents were not significantly different at baseline. However, baseline drug abstinence self-efficacy among African Americans was significantly higher than among Latinos, and this is consistent with prior research. Although abstinence is related to higher levels of abstinence self-efficacy [18,20,21], abstinence may not be the only indicator of successful long-term recovery. Other environmental factors, such as the social, family, medical, and employment aspects may be impacting Latinos’ self-efficacy to abstain from drug use. Regarding how this knowledge can be applied to routine clinical practice, it may be necessary for treatment providers to follow-up with Latino recovering recovery after being discharged from treatment in order to track their functioning and environmental stressors.

## Figures and Tables

**Table 1:** Lifetime substance use in years.

	Sample	Latino	Caucasian	African American	Statistical Significance
	*Mean (SD)*	*Mean (SD)*	*Mean (SD)*	*Mean (SD)*	
Alcohol (to intoxication)	11.32 (9.31)	8.73 (8.63)	13.77 (10.12)	11.46 (8.68)	
Alcohol (any)	16.21 (8.49)	15.33 (8.77)	17.17 (9.14)	16.13 (7.62)	
Heroin	2.5 (6.4)	5.0 (9.4)^c^	1.3 (3.3)	1.1 (4.1)^c^	*
Methadone	.37 (1.3)	.53 (1.4)	.27 (1.0)	.31 (1.5)	
Other opiates/analgesics	1.0 (3.7)	.50 (1.4)	1.8 (4.9)	.76 (3.7)	
Barbiturates	1.2 (4.1)	1.0 (3.0)	1.7 (5.2)	.90 (3.7)	
Sedatives/hypnotics/tranquilizers	1.4 (4.2)	.77 (2.3)	2.7 (5.6)	.71 (3.8)	
Cocaine	7.9 (7.3)	6.3 (7.3)	7.3 (6.7)	10.2 (7.6)	
Amphetamines	3.0 (5.4)	2.2 (4.6)	5.2 (6.4)^a^	1.4 (4.3)^a^	*
Cannabis	10.7 (9.3)	8.0 (8.0)	11.9 (9.2)	12.1 (10.3)	
Hallucinogens	2.1 (5.0)	.67 (1.4)^b^	4.5 (7.1)^a b^	1.2 (3.9)^a^	*
Inhalants	1.1 (3.8)	.50 (1.5)	1.9 (5.3)	.79 (3.7)	
More than 1 substance	8.9 (8.3)	7.5 (7.6)	9.2 (8.9)	10.1 (8.6)	

* *p.* < .05, ^a^ Significant differences between Caucasians and African Americans, ^b^ Significant differences between Caucasians and Latinos

* p.<.05, ^c^Significant differences between Latinos and African Americans.

**Table 2:** Baseline and Follow-up Days of Drug Use in the Past 90 days.

	Sample	Caucasian	African American	Latino	Statistical Significance
	*mean (SD)*	*mean (SD)*	*mean (SD)*	*mean (SD)*	
Number of Days of Drug Use in the Past 90 Days					
Baseline	2.52 (12.40)	1.97 (7.60)	5.16 (20.0)	0.42 (1.43)	
One-Year Follow-up	11.14 (28.96)	11.36 (24.61)	3.57 (16.37)	18.85 (40.75)	
*N =* Baseline	93	31	31	31	
One-Year Follow-up	63	22	21	20	

**Table 3:** Baseline and Follow-up Levels of Drug Abstinence Self-Efficacy.

	Sample	Caucasian	African American	Latino	Statistical Significance
	mean (SD)	mean (SD)	mean (SD)	mean (SD)	
Drug Abstinence Self-Efficacy					
Baseline	3.99 (1.04)	3.90 (1.01)	4.24 (.92)^a^	3.85 (1.17)^a^	*
One-Year Follow-up	4.07 (1.24)	3.88 (1.33)	4.29 (1.20)	4.06 (1.20)	
N = Baseline	93	31	31	31	
One-Year Follow-up	62	22	20	20	
